# A Case of Sinonasal Inverted Papilloma Suspected as Postoperative Recurrence of Eosinophilic Chronic Rhinosinusitis

**DOI:** 10.7759/cureus.69971

**Published:** 2024-09-23

**Authors:** Takeshi Tsuda, Kiyohito Hosokawa, Soichiro Fujii, Kansuke Kido, Hidenori Inohara

**Affiliations:** 1 Otolaryngology - Head and Neck Surgery, Osaka University Graduate School of Medicine, Suita, JPN; 2 Pathology, Osaka University Graduate School of Medicine, Suita, JPN

**Keywords:** endoscopic sinus surgery, eosinophilic chronic rhinosinusitis, eosinophilic infiltration, histopathological diagnosis, inverted papilloma, sinonasal tumors

## Abstract

Chronic rhinosinusitis (CRS) is a persistent inflammatory disease affecting the nasal and paranasal sinus tissues, classified into two main categories: one associated with nasal polyps (CRSwNP) and one without them (CRSsNP). A particular form of CRSwNP, known as eosinophilic CRS (ECRS), is distinguished by the excessive presence of eosinophils in the affected tissues. While surgical intervention and corticosteroids are the standard treatments, high relapse rates have led to increasing interest in biological treatments. Inverted papilloma (IP), a benign yet recurrent tumor with potential for malignancy, often complicates diagnosis when concurrent inflammation is present. We present a case of a 56-year-old man with a long-standing history of ECRS, initially suspected to have experienced a recurrence. Imaging suggested the possibility of IP, but biopsy results showed a marked increase in eosinophil levels. Following surgery, the diagnosis of IP was confirmed, and no malignancy was found. A year after surgery, the patient remained free of recurrence. This case emphasizes the difficulty of differentiating ECRS from IP due to their overlapping histological features. To ensure accurate diagnosis, a thorough evaluation combining radiological, endoscopic, and pathological methods is crucial.

## Introduction

Chronic rhinosinusitis (CRS) is an inflammatory disease of the upper airway that affects the nasal cavity and paranasal sinuses. These conditions are broadly classified into two types: CRS with nasal polyps (CRSwNP) and CRS without nasal polyps (CRSsNP) [1]. Eosinophilic CRS (ECRS) is a subtype of CRSwNP, distinguished by marked infiltration of eosinophils within the paranasal sinus tissues [2,3]. ECRS is recognized as a form of type 2 inflammatory airway disease involving various immune cells and molecules [4-6,7]. The symptoms typically include nasal obstruction, purulent nasal discharge, and olfactory disturbances. Furthermore, the disease is frequently associated with bronchial asthma and eosinophilic otitis media. Additionally, the condition is often complicated by non-steroidal anti-inflammatory drug (NSAID) intolerance and tends to be more severe when these comorbidities, such as bronchial asthma and eosinophilic otitis media, are present. Current management strategies for ECRS involve a multifaceted approach that includes surgical intervention and the use of both local and systemic corticosteroids. Especially, endoscopic sinus surgery (ESS) is widely regarded as the primary treatment for ECRS. Particular emphasis is placed on the complete removal of the bony septum within the paranasal sinuses. Despite these interventions, patients frequently experience high recurrence rates. Consequently, biological therapies have been incorporated into treatment protocols for cases resistant to conventional methods. Although many biological therapies exist, anti-IL-4 receptor alpha antibodies and anti-IL-5 antibodies are currently the only ones approved for CRSwNP in Japan.

Conversely, paranasal inverted papilloma (IP) is a benign tumor; however, it has a recurrence rate of approximately 10% after treatment and frequently carries the risk of malignant transformation [8-10]. The primary symptoms include nasal obstruction and discharge, but malignant involvement of adjacent tissues may also lead to visual disturbances. IPs typically present as unilateral formations and exhibit distinct imaging characteristics. However, these masses frequently manifest with inflammatory changes. A definitive diagnosis may be difficult to achieve without submitting an appropriate tissue sample for pathological analysis. The primary treatment is surgical resection; however, it can be combined with radiotherapy or chemotherapy when malignancy coexists.

This report details a case where preoperative biopsies from multiple sites did not confirm the suspected diagnosis of IP, as indicated by imaging studies. The sinus tissue sampled during this examination revealed substantial eosinophilic infiltration, indicating ECRS recurrence. 

This article was previously presented as a poster at the Society of Practical Otolaryngology Meeting on June 28-29, 2024.

## Case presentation

**Figure 1:**
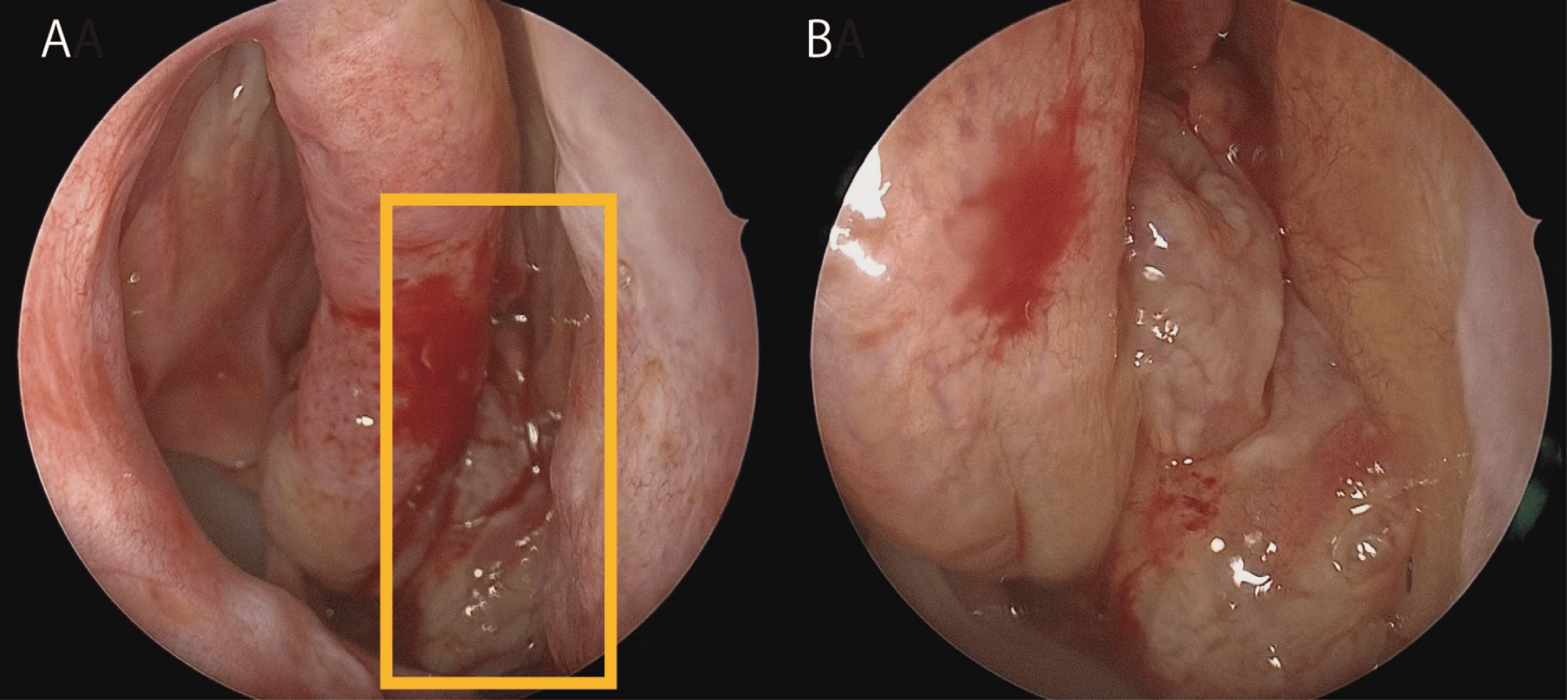
Nasal endoscopic findings at initial presentation (A) Neoplasm (yellow square) originating from the posterior aspect of the right nasal cavity. (B) The mass exhibits focal surface irregularities.

A 56-year-old male initially underwent bilateral endoscopic sinus surgery (ESS) for ECRS at another hospital X seven years ago. Despite discontinuing hospital visits, he presented to hospital X one month ago with a worsened post-nasal drip, prompting a referral to our department by another local doctor due to suspected ECRS recurrence. During the initial assessment, a noticeable mass with surface irregularities was observed in the posterior right nasal cavity, whereas no significant abnormalities were observed in the left nasal cavity (Figures 1A-1B).

**Figure 2:**
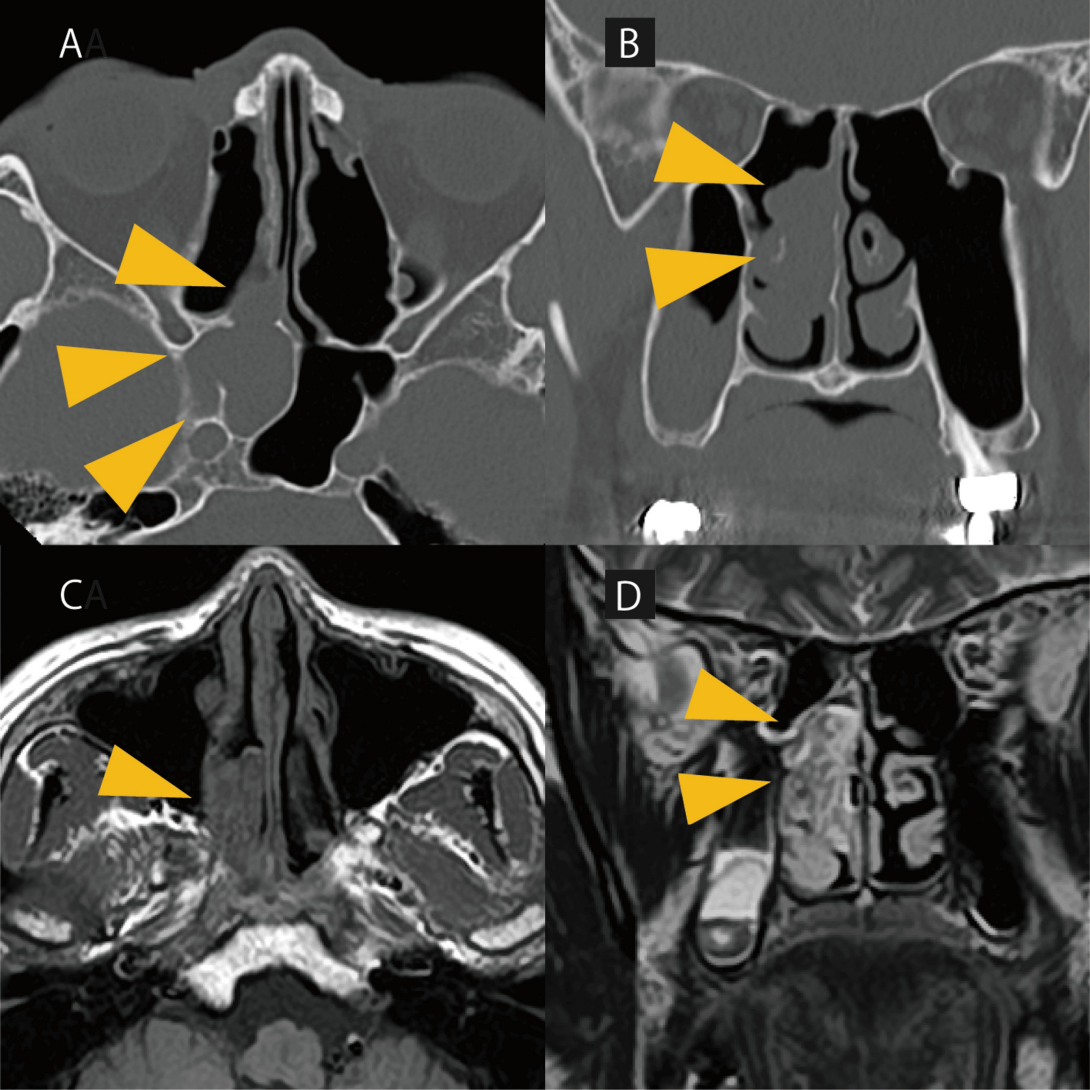
Initial radiological findings (A, B) Axial computed tomography (CT) of the paranasal sinuses demonstrating soft tissue opacification in the sphenoid sinus and its anterior extension. No evident osseous destruction is observed. (C, D) Magnetic resonance imaging (MRI) of the sinuses revealing an isointense mass on T1-weighted images and a heterogeneous iso- to hyperintense mass on T2-weighted images, exhibiting a characteristic cerebriform pattern. Yellow triangles indicate the tumor on both imaging modalities.

Sinus computed tomography (CT) revealed a soft tissue shadow extending from the sphenoid sinus to the nasopharynx, which exhibited a convoluted cerebriform appearance on magnetic resonance imaging (MRI) (Figures 2A-2D). It was difficult to estimate the base of the mass using only CT, as there was no obvious bone thickening either within the mass or at its attachment site. However, based on the MRI patterns, the base was presumed to be slightly lateral to the anterior wall of the sphenoid sinus.

**Figure 3:**
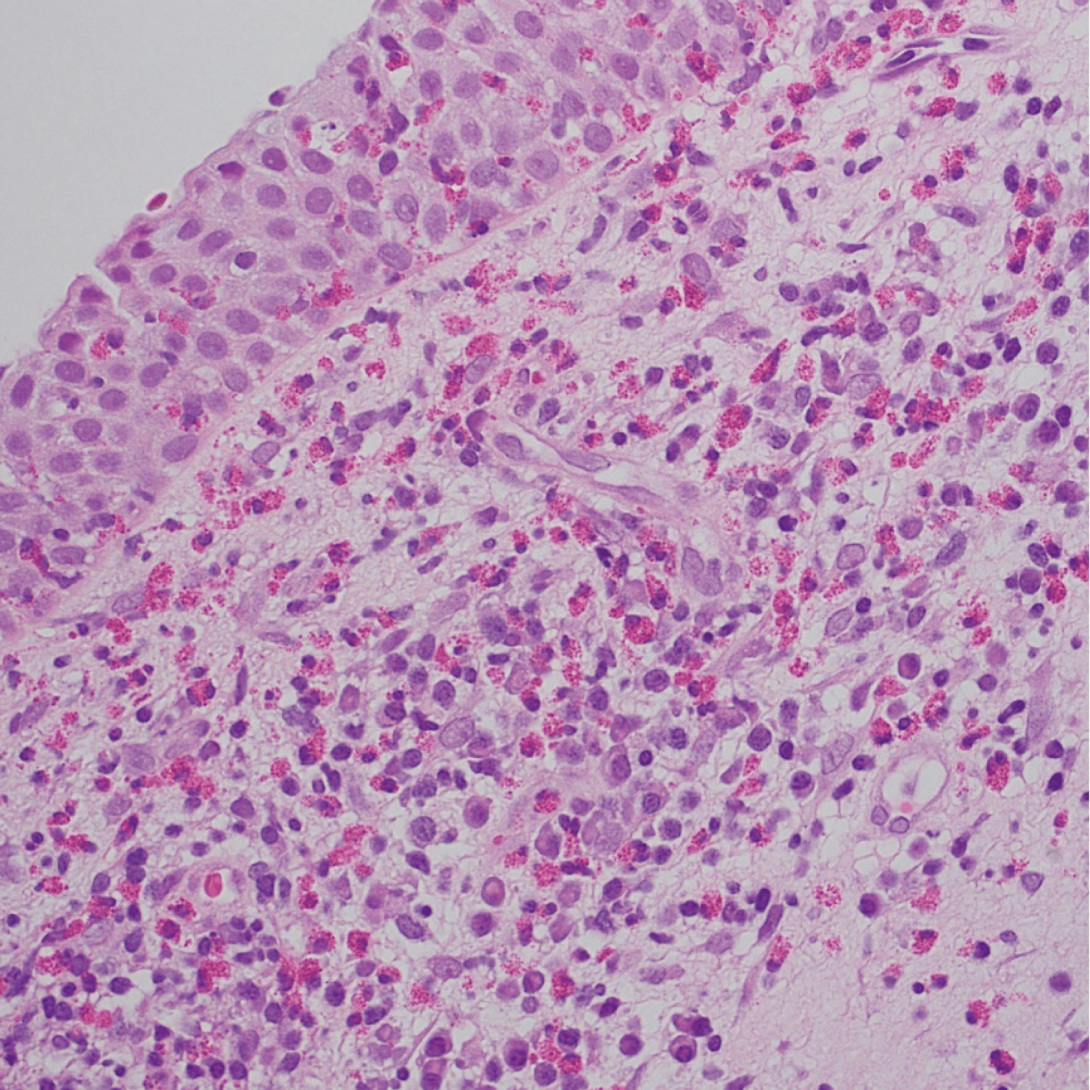
Histopathological findings from the outpatient biopsy Microscopic examination reveals marked eosinophilic infiltration.

Multiple biopsies from the apical site and posterior site were performed under the suspicion of an IP; however, no clear neoplastic lesions were detected. The tissue eosinophil count was significantly elevated, indicating ECRS recurrence (Figure 3).

**Figure 4:**
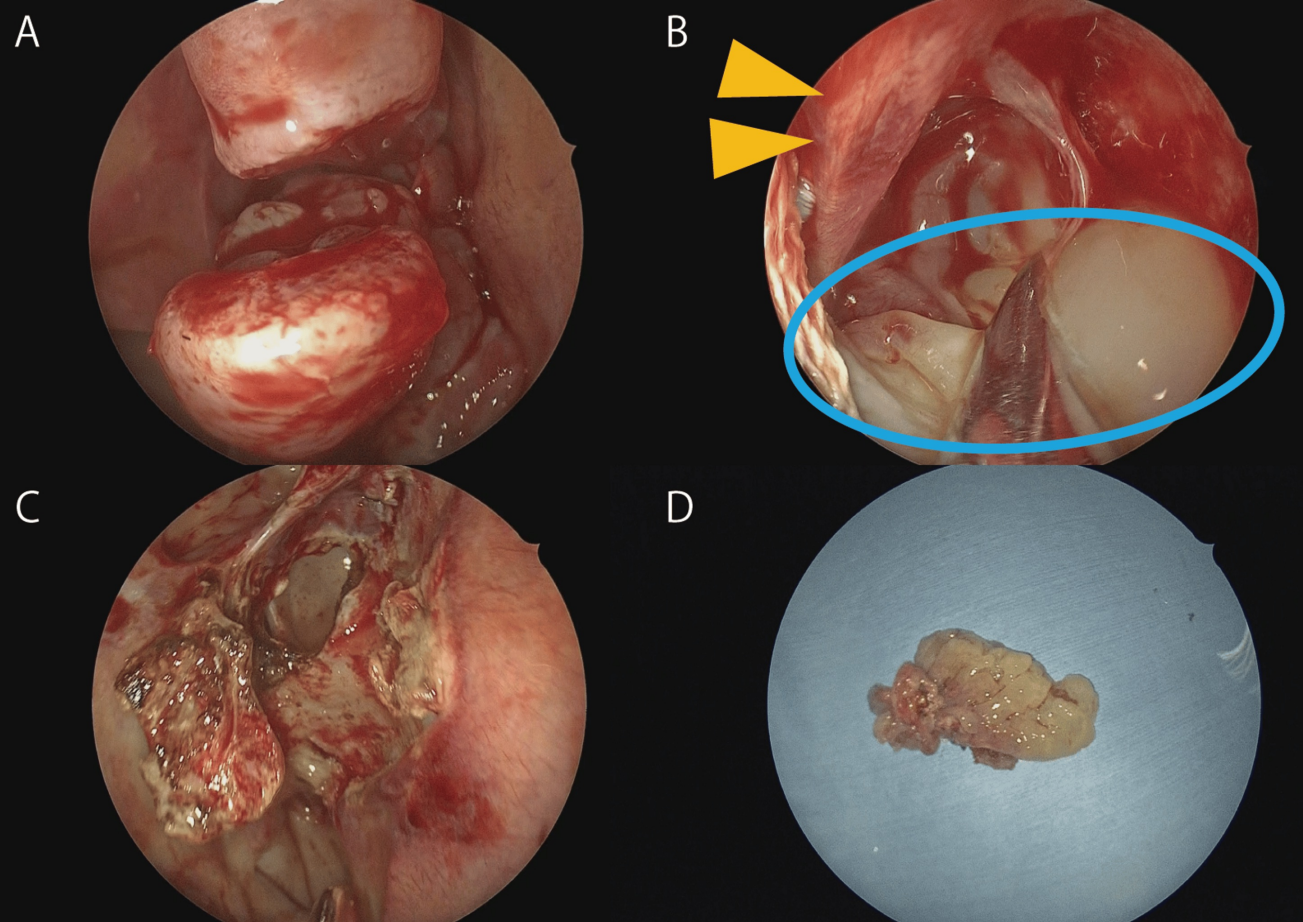
Intraoperative findings (A) Division of the middle turbinate enabling anterior visualization of the neoplasm. (B) The lesion (light blue area) demonstrates broad attachment to the inferior aspect of the superior turbinate and the anterior wall of the sphenoid sinus. The yellow triangle indicates the superior turbinate. (C) Post-excision view demonstrating removal of surrounding mucosa and underlying bone at the lesion base. (D) Enbloc resection specimen of the inverted papilloma.

Subsequently, right endoscopic sinus surgery was conducted under general anesthesia X for +6 months thereafter. The mass originated broadly from the anterior wall of the sphenoid sinus and was resected en bloc from the base after the removal of the lower end of the upper turbinate (Figure 4).

**Figure 5:**
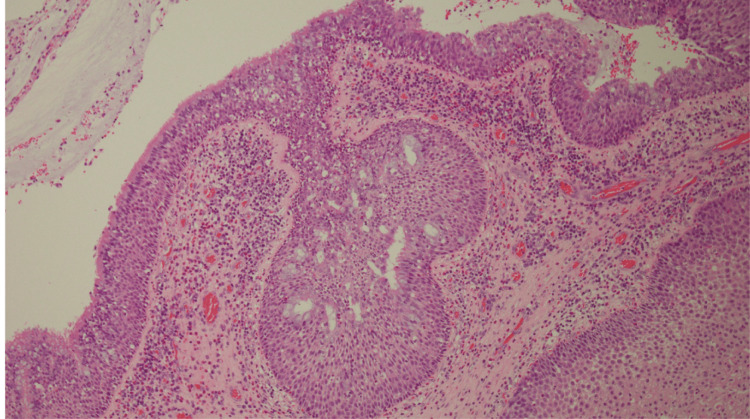
Histopathological findings of the surgical specimen Microscopic examination reveals features consistent with inverted papilloma, characterized by endophytic growth and epithelial invagination, without evidence of malignancy.

Definitive histopathological analysis of the resected tissue confirmed the presence of IP without malignancy (Figure 5).

The patient is currently undergoing outpatient surveillance one year postoperatively and shows no signs of recurrence.

## Discussion

ECRS is an inflammatory condition that affects the upper respiratory tract and is characterized by a high rate of recurrence [2,3,7]. Conversely, IP is a benign neoplasm of the sinonasal tract that has a high recurrence rate and possesses malignant potential, thus requiring meticulous management [11-13]. Although these conditions share similar recurrence patterns, they are distinct pathological entities. To date, no study has demonstrated an increased incidence of IP in patients with ECRS.

The primary therapeutic approach for both conditions is surgical intervention, although different techniques are employed for each. IP surgery requires complete excision of the neoplastic lesion, including the base and adjacent mucosa, whereas ECRS surgical management focuses on mucosal preservation. Consequently, accurate preoperative diagnosis is of paramount importance. Although biopsy is the standard procedure for definitive diagnosis, it may yield false-negative results depending on the sampling site [14]. In this case, multiple biopsies were performed due to the suspicion of IP based on endoscopic findings and growth patterns. However, a conclusive preoperative diagnosis remains elusive. Histologically, IP frequently exhibits significant eosinophilic infiltration, which potentially complicates the differentiation from ECRS specimens in certain regions [15].

In the present case, as previously noted, extensive eosinophilic infiltration in outpatient biopsies initially suggested ECRS recurrence. However, a comprehensive clinical evaluation necessitates an alternative surgical approach. The decision-making process incorporated several factors, including (1) the unilateral nature of the lesion, (2) focal irregularities on the surface, and (3) the inability to exclude papillomatous patterns based on radiological findings. Consequently, the surgical approach involved an en bloc resection of the lesion, including its base. The postoperative course was uneventful. Six months after the intervention, mild granulation tissue was observed; however, at the one-year follow-up, no evidence of tumor recurrence was observed. The patient was scheduled for regular outpatient evaluations to facilitate early detection of potential recurrence or malignant transformation.

Despite the absence of papillomatous structures in previous surgical specimens from other institutions, it is noteworthy that the middle meatus is typically the preferred site for definitive ECRS diagnosis. Comprehensive examination of all polypoid lesions may not always be performed, potentially leading to diagnostic inaccuracies even if IP was present during the initial operation. Furthermore, the broad nature of the lesion complicates the distinction between de novo occurrences and recurrences.

## Conclusions

We present a case initially presumed to be recurrent ECRS based on preoperative assessment but was ultimately diagnosed as IP upon definitive histopathological examination. This case illustrates the potential for false-negative results in IP diagnosis, even with multiple-site biopsies, due to variability in sampling sites. Additionally, the presence of marked eosinophilic infiltration in some cases of IP can lead to misdiagnosis if it relies solely on histopathological findings. Consequently, a comprehensive diagnostic approach that integrates radiological and endoscopic findings with histopathological findings is crucial for accurately differentiating these entities.
